# Improving care for immigrant women before, during, and after childbirth – what can we learn from regional interventions within a national program in Sweden?

**DOI:** 10.1186/s12913-022-08054-7

**Published:** 2022-05-17

**Authors:** M. E. Nyström, E. C. Larsson, K. Pukk Härenstam, S. Tolf

**Affiliations:** 1 Department of Learning, Informatics, Management and Ethics, Medical Management Centre, Karolinska Institutet, 171 77 Stockholm, Sweden; 2 Department of Public Health and Clinical Medicine, Epidemiology and Global Health, Umeå University, 901 87 Umeå, Sweden; 3 Department of Global Public Health, Karolinska Institutet, Stockholm, Sweden; 4 Department of Women’s and Children’s Health, Karolinska Institutet, 171 77 Stockholm, Sweden; 5 Department of Women’s and Children’s Health, Uppsala University, Uppsala, Sweden; 6 Pediatric Emergency Department, Astrid Lindgren Children’s Hospital, Karolinska University Hospital, Stockholm, Sweden

**Keywords:** Immigrant women, Healthcare improvement, Complex interventions, Sexual and reproductive health, Antenatal care, Delivery care, Equal care, Empowerment

## Abstract

**Background:**

Migration has increased the number of immigrant women in western countries, which has led to a need to adapt sexual and reproductive health (SRH) care to a larger variety of experiences. Examples of problems are poor access/utilization of SRH services among migrants and a comparatively higher rate of mortality and morbidity in relation to pregnancy, especially among those from low- and middle-income settings. Attempts to improve SHR care must consider the complexity of both the problem and the system. A national program to improve women’s health in Sweden provided opportunities to study interventions aimed at immigrant women, using a complexity theory lens. The purpose was to explore the characteristics and complexity of regional interventions aiming to improve care and health of immigrant women before, during and after childbirth, and provide knowledge on how regional healthcare actors perceive and address problems in these areas.

**Methods:**

This archival research study is based on qualitative data from detailed yearly reports of all regional program interventions (*n* = 21 regions) performed between January 2017 and January 2019. The archival data consists of the regional actors’ answers to an extensive questionnaire-like template, where the same questions were to be filled in for each reported intervention. Data analyses were performed in several steps, combining classic and directive content analysis.

**Results:**

Six problem categories were addressed by 54 regional interventions, 26 directed at immigrant women and their families, 11 at healthcare staff, and 17 at the organizational system. The simple level interventions (*n* = 23) were more unilateral and contained information campaigns, information material and translation, education, mapping e.g., of genital mutilation, and providing staff and/or financial resources. The complicated interventions (*n* = 10) concerned increasing communication diversity e.g., by adding iPads and out-reach visits. The complex interventions (*n* = 21), e.g., health schools, integration of care, contained development, adaptions, and flexibility with regards to the immigrant women’s situation, and more interaction among a diversity of actors, also from the wider welfare system.

**Conclusions:**

It is important that complex problems, such as ensuring equal care and health among a diverse population, are addressed with a mix of simple, complicated, and complex interventions. To enhance intended change, we suggest that pre-requisites e.g., communication channels and knowledge on behalf of immigrant women and staff, are ensured before the launch of complex interventions. Alternatively, that simple level interventions are embedded in complex interventions.

## Introduction

The number of immigrant women in western countries, including Sweden, have increased due to global migration (here broadly defined as being born in one country and having moved to another country) [1]. Hence, in the receiving countries, the proportion of women with a migrant background in need of sexual and reproductive health (SRH) care services, such as contraceptive counselling, antenatal care, and care during delivery and the post-partum period, has also increased due to increased migration [2]. Immigrant women is a heterogenous group with very different backgrounds, knowledge, experiences, and needs, and the healthcare system needs to be able to meet this variety [3]. At the same time, being new in a certain context is associated with some common experiences. Access to general preventive health services among immigrants in Europe, especially among migrants from low-and middle-income settings outside of EU, is lower than the access in the general population [4]. A review summarized the challenges for immigrant persons to access general healthcare in three themes: communication, confidence, and continuity of care [5]. Research from high-income countries, including Sweden, has shown similar reasons e.g., that the poor access/utilization of SRH care services among immigrant women is due to language barriers, cultural factors, and lack of knowledge about the healthcare systems [6–8]. Immigrant women experience mortality and morbidity more often in relation to pregnancy as compared to native born women [9, 10]. In a review of pregnancy related care the access was connected to: 1) challenges to navigate in a new healthcare system - including physical access and lack of trust in the new system; 2) lack of understanding - language problems, lack of appropriate information, and cultural differences; 3) how healthcare providers meet women’s needs – e.g. quality of care, attitudes, trust and continuity of care; and 4) other factors complicating the women’s’ lives e.g. trauma, financial challenges, lack of social/relationship support [11]. Higher abortion rates and lower contraceptive use among immigrant women in Sweden and other European countries have also been reported [12–16].

Several knowledge gaps have been identified regarding strategies for how healthcare is best provided to immigrant women in western countries. For example, midwives in abortion care have illustrated how the challenges of providing care to immigrant women is due to them feeling unsure of how to best cater for them [7]. There are studies on interventions for improving SRH care for immigrant women and their effects. A study in Denmark found that the use of printed information materials and a mobile application improved communication between healthcare providers and pregnant immigrant women [17]. Person-centered care, high-quality care, and continuity of care that incorporates aspects of cultural competency and trauma aware care have been proposed as ways to address social determinants of health and reduce discrimination against immigrant women [11]. To understand how to achieve medical and health-related outcomes however, process measures and intermediate outcomes must also be studied, for example how better communication, learning, and involvement among and between staff and immigrant women can be achieved (e.g., [18, 19]).

A large national state initiative 2015–2022 to improve women’s health in Sweden’s decentralized healthcare system provided an opportunity to investigate interventions targeting immigrant women. The initiative aimed to improve all women’s health and to promote equal health mainly by improving antenatal, delivery, postpartum, and neonatal care. This national soft law initiative (e.g., [20]) is aimed at improving women’s health and care provided before, during and after childbirth. It is based on an agreement (AG) between the government and the Swedish Association of Local Authorities and Regions (SALAR).

In 2015, 17% of the population in Sweden was born in another country, with the largest groups born in Syria, Iraq, and Iran [21]. The percentage has since increased, especially after the war in Syria where Sweden accepted many refugees. In 2021 the ten largest groups of migrants in Sweden were (in order): Syria, Iraq, Finland, Poland, Iran, Somalia, Afghanistan, former Yugoslavia, and Bosnia-Herzegovina [21]. In Sweden decisions on health services are delegated to 21 regional self-governing regions, each with a responsibility for healthcare services for their population. Regional councils are elected by popular vote among residents and mainly funded by regional tax. Maternal healthcare, such as antenatal care (ANC), and contraceptive counselling is provided at outpatient maternal healthcare clinics that are led by midwives. A pregnant woman usually does not see a doctor during her pregnancy, unless there is a health problem. Moreover, women are offered routine postpartum care within 6–16 weeks after giving birth [22]. There are various ways that the independent regions and the regional actors involved in the program can choose to design and deliver interventions aimed to change the current situation. Thus, a study of this national program can provide more knowledge, not only on how care is provided to immigrant women in Sweden, but specifically on what measures in of identified improvement areas described above that are implemented nation-wide in a decentralized healthcare system.

The success of interventions to improve care is dependent on their consideration of the complexity of the problem addressed as well as the degree of complexity in the targeted system [23–25]. A simple system has fewer agents and components while a complicated system has more agents and components, both characterized by their well-defined interrelations. In these types of systems, the overall system behavior is predictable. Complex systems on the other hand have ill-defined, adaptive boundaries and consists of agents interacting with other systems and agents based on less predictable internal rules, and in ways that co-evolve and change contexts and other systems [24]. There is a risk of oversimplifying interventions and of underestimating the complexity in which healthcare actors and organizations exists, which can increase the risk of early rejection and abandonment, for example when applying technological solutions [26–28]. This highlights the importance of taking several parts of the system, the interaction between them, and their level of complexity into consideration when introducing an intervention [29].

Glouberman and Zimmerman also view healthcare systems as complex and see it as a complex problem to improve them, while attempts to intervene mostly treat them as merely complicated [25]. They discuss the distinction between different type of problems in terms of their complexity. Simple problems contain basic issues of technique and terminology, and when they are mastered followers can use the recipe and be assured of success. Complicated problems contain subsets of simple problems but cannot be reduced to an assembly of simple problems [25]. Their complicated nature relates to the scale of the problem, coordination, and specialized experts needed. Complex problems include both complicated and simple problems, but also require the understanding of unique local conditions [25]. Thus, in Glouberman and Zimmerman’s distinctions the complexity of a problem is related to how it needs to be addressed (intervention characteristics). Other definitions of intervention complexity highlight their degree of flexibility and non-standardization [30], or the number, variability, and flexibility of: its interacting components; the behaviours required; the groups or organizational levels targeted; the outcomes; and the flexibility of the tailoring permitted [31].

To be successful in interventions targeting the immigrant women, with the variety this group represents, it can be argued that the solutions need to match the degree of complexity of the problem addressed. To use a simple solution to a complex problem may not result in the intended outcome (e.g., [26]). The need to move beyond an over-reliance on individual-level theorizing and to better understand public health interventions in their context when the aim is to achieve population-level change has also been highlighted as important for the future direction of intervention research in public health [32]. Achieving equal care and health for immigrant women represent such complex problem and interventions addressing it is launched within the rather complex healthcare system in Sweden. Addressing the complexity of problems and change interventions has been done in other studies, for example in Obstetrics and Gynecology care in Denmark [33]. Such approach can provide insights that may aid the planning of interventions to improve SRH services for immigrant women.

Accordingly, the purpose of the study was to explore the characteristics and complexity of regional interventions aiming to improve care and health of immigrant women before, during and after childbirth, and provide knowledge on how regional healthcare actors perceive and address problems in these areas. The more specific aims where to investigate 1) the perceived problems in providing care for immigrant women the regional healthcare providers choose to address; and 2) the nature of the interventions they launched to improve and promote equal health and equal care for immigrant women before, during and after childbirth. The first aim can provide an insight into how problems and challenges are perceived by key actors and the second into the characteristics of the interventions launched to address them.

## Methods

### Study design

This archival research study [34] is based on qualitative data providing texts and figures in the form of detailed yearly activity reports from each one of Sweden’s 21 regions, designated to SALAR and other key actors in the national program. The archival data consists of the region’s answers to an extensive questionnaire-like template form. The Excel based template has several sheets and provide opportunities to write as much as judged as needed for others to understand the activities that has been going on, why they were initiated, their details, and perceived effects. The template questions used in this study is presented in Table 1. The data has been collected from the regions by SALAR and as researchers longitudinally studying the national program, we were given access to this data base. Due to the principle of public access to information applied to public organizations in Sweden the activity reports are also openly accessible from the regions. The study focuses on understanding a sub-process in a complex intervention program (i.e., the types and complexity level of the interventions chosen by the healthcare regions to address needs and problems), inspired by the on-going discussion of the need for more process evaluations (e.g., [35]). It does not focus on the entire program, but on a subgroup of interventions related to one of the program goals and directed at a subgroup of women i.e., immigrant women.

**Table 1:** Template for the regions (*n* = 21) yearly activity report - to fill in for each on-going or completed intervention

The name of the intervention:	
***A) Description of the purpose, goal/s and expected results of the intervention***	
1. Briefly describe the purpose of the intervention	
2. Describe the goal of the intervention. What results should be achieved and when?	
***B) Description of activities that has been realized as part of the intervention, and what part/s of the organization they concern, and the focus of the intervention***	
1. Which activities has been realized?	
2. What part/s of the organization does the intervention concern? *Mark one or several alternatives.*	a) Maternal Health Care (MHC) including specialist MHC
	b) Delivery clinics/maternity wards
	c) Neonatal care
	d) Primary healthcare - other than MHC
	e) Open specialist care - other than specialist MHC
	f) Other inpatient care
	g) Other
3. Does the intervention have a particular focus on these target groups? *Mark Yes or No.*	a) Women born in another country
	b) People with low education level
	c) People exposed to violence
	d) Socio-economically vulnerable (geographical) areas
	e) People with mental illness
	f) The partner to the women giving birth
***C) Description of the underlying needs that the intervention is based upon***	
1. Based on what need/s where the intervention selected?	
2. How was the need/s identified?	
***D) Timeframe of and finances for the intervention***	
1. Start - *Specify year, month*	2. Finish - *Specify year, month*
3. Amount financed by the national program? *Specify in 1000 SEK*	
4. Is the intervention completed? *Yes or No – if Yes continue to F to describe results of completed interventions*	
***E) Description of the results so far in on-going interventions***	
1. What results/effects can you see so far?	
2. How has these results/effects been measured - alternatively how is/are the results/effects planned to be followed-up?	
3. If feasible, describe shortly any special effects on the following groups	a. Women born in another country
	b. People with low education level
	c. People exposed to violence
	d. Socio-economically vulnerable areas
	e. People with mental illness
	f. The partner to the women giving birth
***F) Description of the results of completed intervention***	
1. Describe shortly results/effects of the completed intervention	
2. How has these results/effects been measured?	
3. *(same as QE3 above)*	
***G) Reflections over completed interventions***	
1. Has the intervention led to any un-foreseen consequences?	
2. What potential results/effects is expected in a longer time perspective?	
3. Is there plans/preparations for how results shall be treated and sustained within the organisation? *Yes/No/Partly*	
4. If Yes or Partly on QF6 – describe how	
5. What is the most important learning from this intervention – if you should do a similar intervention again?	
***H. Other comments***	
1. Any other comments?	

### Study setting - the national program to improve delivery care and women’s health in Sweden

The Swedish healthcare system is mainly tax-funded and decentralized compared to many other countries. The government can initiate and fund national initiatives through agreements (AG) with all regional authorities, via their coordinating member organization SALAR. Such agreements enable coordinated interventions on national, regional, and local levels and this national program (AG 2015–2016; 2017–2019) is one example. A national team at SALAR has the assignment to coordinate, support and follow-up the regional interventions within the scope of the program. The program started late Autumn 2015 with a preparation period, and it runs until 2022. It has five overarching goals: to increase 1) person centered care, 2) patient safety, 3) equal care, 4) evidence-based care, and 5) access to care. The total amount of funding for the program 2015–2022 is 8,9 billion SEK, the main part distributed directly to the regions based on population size. The self-governed regions are then free to decide how to use these funds based on regional and local needs and situations, if the decisions, activities, and use of the funding align with the overarching goals of the program. The Swedish Agency for Health and Care Services Analysis have the mission to analyze and evaluate the program.

During the program period complementary agreements have been made with slightly different focusses. In one of the earlier agreements (AG 2017–2019) there was a special focus on interventions aimed at improving the health of women in socioeconomic exposed situations and areas. One emphasized group was immigrant women in general, but also women arriving in Sweden from warzones in different countries.

### Data collection

#### Yearly regional activity reports

The national program team at SALAR has developed a questionnaire-like report template for the regions’ yearly report to national levels. The template, in the form of an Excel file with several sheets, consists of questions regarding the regional activities aligned to the program each year (see Table 1). To receive the funding, the region representatives were obliged to fill in the template once a year. The archival data in this study consists of the yearly reports of all the interventions targeting women within the program in 2017–2018 reported in February 2019 by all 21 regions in Sweden. The template consists of the following themes for each activity to be qualitatively described: name of the intervention, purpose and aim of the intervention, description of what was done, which organization/providers the activity refers to or includes, what needs or challenge the activity relate to, time frames and funding, results of ongoing intervention, results for completed intervention, future planned activities, and an open category of “other information” (Table 1). The Excel file was first filled in by representatives from each region in 2017, and then new information on new, ongoing, and completed interventions was added for each year.

### Data analysis

An iterative approach using both classic content analysis and directive content analysis [36] was applied. The data analysis was performed in five steps based on the mandatory yearly regional activity reports.

Firstly, all activities reported were scrutinized by one researcher (ST) to select those focusing on the target group immigrant women. The first selection criterium was based on the regional representatives themselves filling in the target group “women born in another country” in the report template (Table 1, question B3). In all, the regions reported 747 activities. Out of these interventions, 93 was marked as having a special focus on immigrant women. Seventeen of the 21 regions filled in interventions targeting immigrant women.

Secondly, one researcher (ST) read through all 93 interventions and selected those interventions that explicitly, manifest, in text, mentioned the target group immigrant women. Interventions concerning “all women” and hence also immigrant women but not them specifically, were not selected. Interventions concerning both “women born in another country” and “women in socio-economically vulnerable areas”, but with no manifest link to immigrant women were not selected. In all, 54 interventions were selected for analysis after the above criteria. The number of interventions per regions were also calculated (Table 2).

**Table 2:** Regional interventions (2017–2018) exclusively directed at immigrant women, and their level of complexity

Region	Population (2019)	Number of interventions judged to be exclusively targeting immigrant women	Simple interventions	Complicated interventions	Complex interventions
A	> 1,000,000	10	6	2	2
B	200,000–300,000	7	3		4
C	300,000–500,000	6	1		5
D	300,000–500,000	6	2	2	2
E	200,000–300,000	4	2	1	1
F	200,000–300,000	3			3
G	200,000–300,000	3	1		2
H	200,000–300,000	3	1	2	
I	< 200,000	2	1	1	
J	200,000–300,000	2	1	1	
K	> 1,000,000	2	1	1	
L	200,000–300,000	1	1		
M	300,000–500,000	1	1		
N	300,000–500,000	1			1
O	> 1,000,000	1	1		
P	300,000–500,000	1			1
Q	< 200,000	1	1		
R	200,000–300,000	0			
S	200,000–300,000	0			
T	200,000–300,000	0			
U	< 200,000	0			
TOTAL: 21	10,373,063	54	23	10	21

Parts of the text describing the 54 interventions (Table 1, questions A1–2, B1, C1–2, E1–3, F1–3, G1–5), was then categorized into four categories inspired by theories of change models [37]; text describing the problem/need addressed, the type of interventions, the output, and the outcome (Table 3). Text describing the problems/needs from each intervention were sorted into themes describing the type of problem addressed (Table 2).

**Table 3:** Problems addressed by the regional interventions

	Problems addressed by the regional interventions	Number of interventions
1.	Low access to, knowledge and utilization of the Swedish healthcare system among immigrant women	14
2.	Higher risk for morbidity and mortality in relation to pregnancy and delivery for immigrant women	9
3.	Lack of resources and methods to support immigrants with special needs	9
4.	Women exposed to or at risk of being exposed to genital mutilation	8
5.	Lower levels of knowledge about health issues and self-managed care regarding SRH among immigrant women (especially asylum seekers)	8
6.	Information provided by healthcare is not offered in all relevant languages	6
		Total: 54

In a third step and based on the same questions as above, the text for each intervention (descriptions of intervention, output, and outcome) was sorted into categories based upon which actor and/or system level the interventions were targeting, i.e., the women (patient), the healthcare staff (at clinic/unit level), or the organizational level and beyond.

Finally, we used the lenses of complexity theory to judge the level of complexity of the described problems and interventions using the text and information from all questions in the template. We used Glouberman and Zimmerman’s [29] distinctions, which connects problems to how they should be addressed (interventions). We used the following definitions of the degree of complexity: 1) simple problems and interventions are characterized as straightforward and predictable with few components, e.g., known problems with few components that can be addressed by following a recipe or checklist; 2) complicated problems and interventions as somewhat predictable but with multiple interacting components and issues, e.g., problem is complicated but predictable and solving them will need specific solutions for the parts but the main process is known, such as when building a new rocket; and 3) complex problems and interventions are characterized as dynamic, unpredictable, not easily divided into constituent components, e.g., all skills needed to address the unpredictable and changing situations that can occur when raising a child (Table 3). Complex interventions are in their nature interactive and dynamic, involve multiple actors, multiple organizations and/or levels, and take the complexity of the problem and system into account. In this study it is the service providers views that are represented in the data (i.e., as formulated by representatives from the 21 regions in Sweden that answered the questions in the template related to each activity reported). It is their descriptions of needs and problems, their description of choices and design of interventions.

In Step 1–2 one researcher (ST) performed the initial analyses and MEN and ECL double checked the categorizations independently, and all three researchers finally discussed and agreed on the naming and content of the categories. In Step 3 ST did the categorization and MEN double checked. The classification into the three complexity categories in Step 4 was performed by ST and MEN independently and then compared and agreed on. Most classifications in Step 4 by these two researchers were the same. In the few cases (*n* = 2) when a disagreement was identified we went back to the original text and discussed our interpretations of the text until consensus was reached. ECL also participated in this discussion.

## Results

In all, 17 out of 21 regions described one or more interventions exclusively targeting immigrant women. Nine regions reported one or two interventions, four regions reported three or four interventions, and four regions reported 6–10 interventions (Table 2). Some regions reported many joint interventions as one intervention while others reported each, sometimes minor, interventions separately. This dissonance between ways of reporting indicates that the number of interventions in Table 2 should be interpreted with caution. The findings are not to be interpreted as describing all that what was done for immigrant women in maternity and delivery care in 2017–18, it merely describes what was financed by the program, and the information collected by a regional program coordinator.

Four regions were responsible for 54% (*n* = 29) of the interventions (*n* = 54) targeting immigrant women in 2016–2018. Out of these, three were urban regions (Table 2) and one was not. Several urban regions and two of the three most populated regions in Sweden reported few interventions, which partly can be explained by the variation in how the regions reported and described their interventions.

### Description of problems and needs addressed

We identified six categories of problems and needs (Table 3) that the selected interventions directed at immigrant women aimed to address (Table 4). In our labeling of the categories, we stayed close to the reported text to represent the region’s perceptions of the problems and needs. The *first category* (*n* = 14) concerned immigrant women’s *lower access to and utilization* of the Swedish healthcare system (includes care services, knowledge, and other types of resources offered) than native Swedish women. For example, some regions reported that women with immigrant background more often than native women seek emergency care instead of primary care, which is the preferred way in Sweden to get the best preventive and safe maternal care. One region reported that research had shown that women from socio-economic areas more often do not come to their cervical cancer screening test, and this was why they had chosen an intervention to increase participation. The category was reported by 11 regions.

**Table 4:**
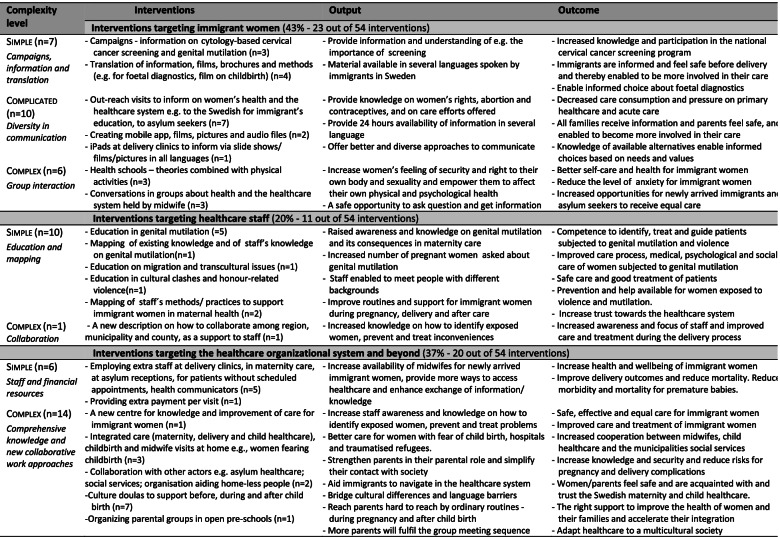
Interventions, outputs, and outcomes as described by the regions and judged intervention complexity level

The *second category* (*n* = 9) concerned a perceived *higher risk for morbidity and mortality* in relation to pregnancy and delivery for immigrant women, identified by seven regions. Several regions referred to statistics from the National Board of Health and Welfare that show that women from overseas countries have worse birth outcomes, compared to native-born women. Others referred to a thesis that had shown that maternal mortality is higher among foreign-born people.

The *third category (n = 9)* was reported by five regions and concerned *lack of resources and methods to support immigrants* with special needs. Examples of special needs addressed were longer appointments for example due to the use of interpreters (extra reimbursement per appointment) and the need to increase healthcare staff’s knowledge and competence regarding migration and transcultural issues.

The *fourth category (n = 8)*, identified by six regions, concerned women exposed to or in risk of *genital mutilation.* The interventions concerned the problem of a high level of women that were at risk or had been exposed to genital mutilation, and a lack of knowledge and a low awareness from healthcare staff on how to prevent and to treat injuries caused by genital mutilation, but also how to approach the patient group to give good care. Several regions described how genital mutilation could cause complications during delivery, potentially fatal.

The *fifth category (n = 8)* concerned a perceived high level of health issues and a need for improved knowledge of reproductive and sexual health among immigrant women, mainly due to a perceived *lack of knowledge and lack of self-managed care*. The interventions in five regions addressed the need of supporting immigrant women in increasing their health literacy by arranging “health schools” and information events about health.

The *sixth category* identified that *information* provided by healthcare *was not offered in all relevant languages.* The regions reported that there was lack of brochures and videos on many languages, apart from Swedish and English. The risk described by some was that this could result in unequal access to important information concerning for example preparation for delivery for the affected women and their families. Six interventions within five regions specifically addressed this problem.

The variation in the region’s descriptions of problems and needs and limited text in this part of the template made it difficult to place the problem within a single complexity level (simple, complicated, complex). The descriptions given depends on how one perceives the reasons or contributing factors to the existing needs and problems, and how well this is described in writing in the report template. When judged more broadly, we identified category 6 as being a simpler problem to address, while the other categories contain a larger level of complexity.

### The targets of the regional interventions

The regional interventions to improve care and health of immigrant women were directed at either the immigrant women and their partners, the healthcare staff, or at the healthcare system - and sometimes beyond (Table 4).

Twenty-six of the 54 interventions were mainly *directed at immigrant women and their families*. These interventions concerned information campaigns about for example cancer screening or risks with genital mutilation, development or translation of information material, and out-reach visits to inform on women’s health and the healthcare system e.g., visits to the Swedish for immigrant’s education. Interventions also included arranging group conversations about health and healthcare or arenas for combining both theoretical knowledge and physical activities.

Eleven of the reported interventions were mainly *directed at healthcare staff*. These consisted of basic web educations and courses for staff regarding e.g., genital mutilation, migration, and transcultural issues or new and clarified staff routines for collaborations.

Seventeen of the reported interventions was *directed at the healthcare organizational system and actors outside the healthcare system*. Some interventions consisted of straightforward interventions, such as adding more staff and resources in clinics or in organizations established for asylum immigrants or increasing reimbursement per visit for immigrant women to allow for longer visits. Other interventions targeted several different organizational actors in the care process to increase integrated care, or enhance collaboration, for example by developing new ways to collaborate with social services and open pre-schools. The use of cultural doulas was also deemed as an organizational intervention, seen as an introduction of new functions and a new staff category.

### From simple to more complex interventions

The interventions were categorized according to their level of complexity, i.e., simple, complicated, or complex interventions (Table 4). Of the 54 interventions 23 were categorized as simple, 10 as complicated and 21 as complex. The 54 interventions were grouped so several identical or similar interventions are represented by one overarching intervention category, reducing the material to 21 intervention types, further described in Table 4.

The *simple level interventions* contained campaigns, information and translation, education, and mapping, and providing staff and/or financial resources, aimed at all three target categories equally (7, 10, 6). The *complicated level interventions* were fewer, and all directed at immigrant women. They concerned the use of more diversity in the communication between health providers/staff and immigrant women and their families. Most of the *complex level interventions* (14 of 21) targeted the healthcare organizational system and other actors in the wider welfare system. Six interventions were directed at immigrant women and one at healthcare staff. Two target groups, immigrant women and the healthcare system and beyond, contained more interventions each than the interventions targeting healthcare staff.

When comparing the regional interventions (Table 4) with the categories of problems and needs identified (Table 3) we found that the problems were addressed in different ways. *Low access to, utilization and knowledge of the Swedish healthcare system among immigrant women* was addressed by a mix of simple, complicated, and complex interventions, from information campaigns, new ways of providing information via different media, conversations in groups, out-reach visits (e.g., to Swedish for immigrant’s education premises), collaboration with other actors and introducing new routines and functions (e.g., cultural doulas).

*Higher risk for morbidity and mortality in relation to pregnancy and delivery for immigrant women* was a more diffuse problem as the reasons for a higher morbidity and mortality among these groups can have several causes. The problem can be viewed as being addressed by several interventions, from increasing staff resources, educating staff, providing information to women, introducing group conversations, culture doulas, and health schools.

*Lack of resources and methods to support immigrants with special needs* (especially asylum seekers) was addressed by educating staff, increasing staff resources, and providing extra payment per visit to clinics, viewed mainly as simple level interventions. There were also more complex level interventions like introductions of new units, new collaborations, and new routines.

*Women exposed to or at risk of being exposed to genital mutilation* can be seen as a complicated or complex problem that was addressed by information campaigns to reach immigrant women and by providing education for staff – mainly simple level interventions. There were also mappings of the existence of genital mutilation and of staff’s knowledge about the subject.

*Lower level of knowledge about health issues and self-managed care among immigrant women (especially asylum seekers)* regarding SRH care was mainly addressed by group interventions and health schools targeting these women, which represents complicated or complex interventions.

*Information provided by healthcare is not offered on all relevant languages* was addressed by translating information already available in various materials, such as brochures and films, to many languages. It also contained creating new mobile apps, films, slide shows, pictures and audio-files in these languages and providing iPads for clinics to use when showcasing them. The way the problem is described indicates that it is perceived as a simpler problem addressed mainly by simple and complicated solutions, which might have required new collaborations (more complexity) when creating new ways to communicate. These interventions are also related to the above problem category regarding knowledge of the Swedish healthcare system and lower levels of knowledge regarding health issues and self-managed care.

Most of the interventions were not, at a general level, adapted to each individual women – they were more designed in relation to the type of group the women represented. Even so, the more complex interventions had an inbuilt degree of flexibility.

## Discussion

The challenges for immigrant persons to access general healthcare has been found to relate to communication, confidence, and continuity of care [5], while access/utilization of SRH care services among immigrant women is lower than native women due to language barriers, cultural factors, and less knowledge about the healthcare systems [6, 7]. We found that similar challenges were identified by regional healthcare actors in the Swedish national program to improve maternity care and women’s health.

### Problem identification and chosen interventions – complex and flexible or simple and standardized?

**Fig. 1:**
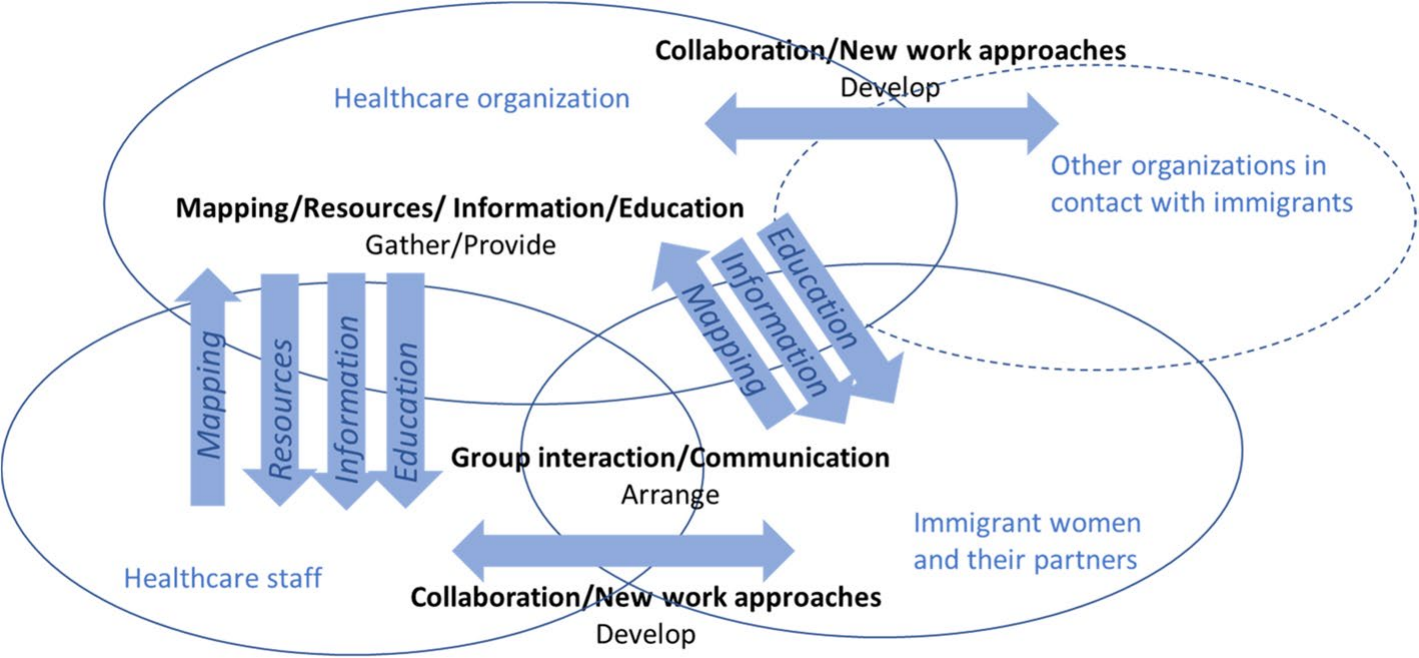
Types of regional interventions in relation to type of interaction, unilateral or joint, among actors within and outside the healthcare system

The interventions chosen to address the problems identified varied in their level of complexity (simple 43%, complicated 18%, complex 39%). The simple, more standardized interventions, such as information campaigns, were to a higher extent unilateral, even though they also could contain degrees of interaction, involvement, and flexibility (Fig. 1). The more complex the interventions were the more development, adaptions, flexibility (regarding the immigrant women’s situation, as individuals or as designated smaller groups), and interaction between different actors were needed. Compared to information brochures or some educational aspects that can be developed in a more standardized way, these complex interventions needed flexibility and an openness for many different situations and actors.

The regions’ choices of more complex interventions also indicate a need for a more patient or person-centered approach on behalf of the healthcare staff and healthcare organization, and a flexibility in collaboration with other organizations outside of healthcare. Complex interventions also contained complicated and simple elements that may become more familiar and less complex over time than during the initial period of development. The choice of using complex interventions may also indicate that there is a more multifaceted view on the problems/needs intended to be addressed by the intervention. In general, complex interventions were used to target complex systems with more actors and different organizations involved.

There were indications of a process - from initially securing a mutual knowledge base that can act as a foundation for further development of the relation between care providers and immigrant women – to more complex and demanding interventions with more interaction. This process could of course be the same for native women, where knowledge also cannot be taken for granted. In general, however, native women have been more exposed to both information and experiences of the Swedish healthcare system and the care provided. This finding highlights questions for further discussions on how to achieve equal care for immigrant women before, during and after delivery and childbirth.

The assumption behind this process or sequence is that more knowledge can increase the capacity to act - in line with the concept of empowerment. Empowerment has been defined as “the capacity of individuals, groups and/or communities to take control of their circumstances, exercise power and achieve their own goals, and the process by which, individually and collectively, they are able to help themselves and others to maximize the quality of their lives ([38], p. xvi). Empowerment is also a concept used in health promotion practices (and research) for individuals to gain greater control over decisions and actions affecting their health [39]. The latter is in line with some of the ambitions of the national program.

Can it be a successful strategy to make sure, with various interventions, that there is enough knowledge (e.g., about healthcare systems and services in Sweden and elsewhere, women’s health and health issues, and actions to promote health) among both staff and immigrant women, to strengthen their abilities to act – and then introduce more complex interventions that require higher involvement of the women, the healthcare staff, and the organization?

If it is anticipated that learning and more knowledge (individually or in groups) can empower the immigrant women to become more involved in their own care, and potentially become more of a co-creator of care, how can learning and empowerment processes be initiated, supported, and sustained? Studies on health promotion activities that focus on empowerment have shown the importance of enabling active learning activities, using visualization tools for self-reflection, and allowing participants to influence activities [40]. Examples of activities that focused on knowledge and learning were described by the regions, for example, the creation of health-schools, centers for knowledge and improvements, new collaborations, new meeting places based on the premises where the women gather, and the use of social groups. These interventions were examples of less standardized approaches. Creating a social environment that strengthens the innate ability by ways of acquiring knowledge, power and experience is what denotes empowerment [41]. There were also interventions to improve information by visualization via films, pictures, and slide shows, sometimes accessible via i-Pads. There was less information on effects of the regional interventions described in the yearly activity reports – even though it was explicitly asked for. If changed behavior of immigrant women is one of the goals then empowerment is expected to provide the individual with greater extrinsic control, intrinsic capacity, self-confidence enabling the individual to overcome external barriers to accessing resources or changing traditional ideology [42]. This output can be measured with subjective data while actual changes in behavior of a group of individuals also can be accessed – but several detected and undetected factors and the mere combination of activities can contribute to changes in the behavior of individuals and groups, making it harder to define what works for whom and when.

To address social determinants of health and reduce discrimination against immigrant women a person-centered, high-quality care that ensures continuity and that incorporates aspects of cultural competency and trauma awareness have been proposed as a solution [11]. This study provides examples on how problems and challenges of providing good care for immigrant women are perceived, and with what types of interventions they are addressed within the Swedish regions. Person-centered care has on national level in Sweden been emphasized as a solution to many problems and challenges encountered, and actively supported nationally (e.g., https://skr.se/skr/halsasjukvard/utvecklingavverksamhet/naravard.6250.html). Most of the complex type of interventions targeted the healthcare organizational system and other actors in the wider welfare system, matching the complex interventions with the complexity of the welfare system in general, and particularly for immigrants. The complex interventions described (and more examples of such approaches) can be seen as attempts to change focus from providing (mainly) standardized care fitting some but not all women – to providing more individual solutions with a more holistic perspective based on a better interaction with and understanding of women in different situations and from different cultural backgrounds.

The interventions described in this study relate to normative advice in previous research, such as the use of interpreters (in this case cultural doulas with a more extensive mission); cross agency working (involving other organizations within the welfare system); respect and accommodate relevant traditional or cultural practices (increasing cultural knowledge among staff) suggested in the review by Fair et al. [11]. The authors highlight the need for more research and further exploration of the needs of different migrant populations as a base for tailored interventions. Complex interventions that involve many actors and that crosses organizational borders presents many challenges. Among other things they rely on people to be able to collaborate and take on cross-boundary spanning challenges and roles and knowledge brokering tasks and strategies (e.g., [43–45]). For key actors with power to make decisions the challenge is to gain an understanding of the complexity of the systems, the problems, and the interventions - so time frames and resources can be tailored to fit both the situation and the intervention.

Here we did not explicitly study the indicators used by the program to assess the outcomes in the program target areas. One example of the national indicators for equal care for immigrant women that is used to indicate progress within the program is the proportion of immigrant women that visited maternity care for a health check after childbirth, which has increased from 69,6% in 2015 to 80,2% in 2019 [46]. Assessing outcomes of improvement interventions in maternity care based on medical or clinical outcomes can however be difficult without an understanding the specific interventions and the process of implementing them [18]. More knowledge of the change process can aid the choices and adaptions of interventions aimed at increasing equal care and health among diverse populations.

### Study limitations

The study is based on the region’s written reports in a document template (Table 1). One limitation is the definition of migrants. In the report template it was not possible to indicate how “migrants” were interpreted by the regions. Although, and as stated in the background, migrants are known to have worse health outcomes and lower access to care, especially for groups coming from low-and middle-income countries with very different health systems compared to the Swedish system. Hence, we believe that the suggested interventions would reach the migrants with less access to healthcare, but also other women. If the interventions, such as translated information material, also reach migrants with good access to care it can be considered as an extra, but beneficial effect. The complexity levels of the interventions were based on the regional healthcare actor’s views and reports, and as such they represent the healthcare systems perspective. The perspective of the beneficiaries of the interventions is not presented, nor available in our data, which is another limitation of the study. Depending on how the process of reporting was designed in each region the information about each intervention could be more or less, detailed. The interventions were by some regions grouped under themes with descriptions of a series or a network of activities and by some described one by one, resulting in a mix of small, medium, and extensive activities. Thus, the number of interventions directed at immigrant women per region and nation-wide should be interpreted with great caution. Also, the interpretation of the target population for the described interventions seemed to differ in the regional reporting when they filled in the target area. To make sure that we used relevant interventions we went through all activities reported from the 21 regions and judged both the interventions marked as targeted immigrant women and those that was not marked for this group - to make sure we did not miss any intervention. To avoid misunderstandings and biases three researchers were involved in the data analysis, and we used both inductive and deductive content analysis in an iterative process – the latter informed by theories of complexity in interventions and problems. It is also important to remember that more general regional interventions targeting all women also benefit the sub-group of immigrant women, as well as recognizing that some regions may already have invested in activities directed at these women before the on-set of the national program.

## Conclusions

The goal of Swedish healthcare is a good health and care based on equal terms for the entire population. This is yet to be reached as there are groups in society that do not fully benefit from healthcare’s services, affecting their health in different ways. There is also a need for more knowledge on how to reach, empower and positively affect the health of groups of people in society that are hard to reach.

This study attempted to provide some additional dimensions on the description of interventions to improve health and care of immigrant women in Sweden from a macro/meso level perspective. Addressing the region’s problem focus, the main target of their interventions, and the complexity level of their chosen interventions helped us further understand the regional care providers action strategies, within the scope of the national improvement program.

We conclude that it is important to make sure that complex problems, such as ensuring equal care and health among a diversity of populations, are addressed with a mix of simple, complicated, and complex interventions and suggest that pre-requisites for the more complex interventions are ensured, e.g., enough knowledge on behalf of both immigrant women and staff and communication channels and arenas, before their launch. Alternatively, simple level interventions can be embedded in complex level interventions in a process that can enhance the intended changes. These are suggestions both for practice when trying to improve the current situation, as well as for researchers interested in further empirical studies. Future studies that can provide more empirical examples of mixed complexity level interventions and to also address their impact can provide more knowledge on how complex problems and needs can be addressed by tailored intervention packages better suited targeted individuals and groups, especially those that are harder for healthcare to reach.

## Data Availability

The datasets (i.e., activity reports in Swedish from all 21 regions) used and/or analyzed during the current study are available from the corresponding author on reasonable request.

## Abbreviations

AG: Agreement between the government and SALAR
SALAR: Swedish Association of Local Authorities and Regions
SRH: Sexual and reproductive health
